# Automated video-based heart rate tracking for the anesthetized and behaving monkey

**DOI:** 10.1038/s41598-020-74954-5

**Published:** 2020-10-21

**Authors:** Mathilda Froesel, Quentin Goudard, Marc Hauser, Maëva Gacoin, Suliann Ben Hamed

**Affiliations:** 1 Institut des Sciences Cognitives Marc Jeannerod, UMR5229 CNRS, Université de Lyon, 67 Boulevard Pinel, 69675 Bron Cedex, France; 2 Risk-Eraser, LLC, PO Box 376, West Falmouth, MA 02574 USA

**Keywords:** Biological techniques, Physiology

## Abstract

Heart rate (HR) is extremely valuable in the study of complex behaviours and their physiological correlates in non-human primates. However, collecting this information is often challenging, involving either invasive implants or tedious behavioural training. In the present study, we implement a Eulerian video magnification (EVM) heart tracking method in the macaque monkey combined with wavelet transform. This is based on a measure of image to image fluctuations in skin reflectance due to changes in blood influx. We show a strong temporal coherence and amplitude match between EVM-based heart tracking and ground truth ECG, from both color (RGB) and infrared (IR) videos, in anesthetized macaques, to a level comparable to what can be achieved in humans. We further show that this method allows to identify consistent HR changes following the presentation of conspecific emotional voices or faces. EVM is used to extract HR in humans but has never been applied to non-human primates. Video photoplethysmography allows to extract awake macaques HR from RGB videos. In contrast, our method allows to extract awake macaques HR from both RGB and IR videos and is particularly resilient to the head motion that can be observed in awake behaving monkeys. Overall, we believe that this method can be generalized as a tool to track HR of the awake behaving monkey, for ethological, behavioural, neuroscience or welfare purposes.

## Introduction

Tracking variations in autonomous responses has proven to be invaluable in the study of complex behaviours and their physiological correlates in non-human primates^1^. These include tracking changes in pupil diameter^2–5^, in skin conductance^6^, social blinks^7^, blink rates^8,9^, nose temperature^10^ and heart rate (HR)^11,12^. HR measure is of particular relevance in diverse cognitive contexts. For example, it has been shown that HR increases when monkeys watch videos with high affective content^13^ and during learning process^14,15^. In spite of this, very few methods currently allow to easily, reliably and non-invasively track HR in awake behaving untrained monkeys. The aim of the present study is to fill this methodological gap.

The classical tools already available on the market to extract heart rate, such as electrocardiograms (ECG) or pulse oximeters, require a direct contact of electrodes or captors with the skin. Indeed, ECG detects changes in voltage generated by the cardiac muscles and requires to place electrodes on a shaved skin. The pulse oximetry method measures oxygen saturation and pulsations of blood thanks to photoplethysmography (PPG). PPG consists in detecting luminosity variations of the skin that are directly related to changes in blood flow^16,17^. It also involves placing a captor on the subject, usually on the fingers. For both these methods, signal quality can deteriorate in time, due to a displacement of the electrodes or the captor. In addition, when dealing with adult human subjects, this may introduce experimental biases due to the fact that subjects possibly becomes aware of the scope of the study. In young infants or animals, placing the captor or maintaining it all throughout the experiment may turn out to be challenging if not impossible. In monkeys, recording HR either involves ECG or PPG under sedation^18–20^ or implanting a telemetry device for HR measure during behaviour^21,22^. PPG recording or HR measures using captors embedded in a wearable jacket is also an option^11,23,24^. However, this requires intensive monkey training and might bias HR measures due to discomfort or stress.

Ballistocardiography has proven efficient in tracking human HR in open field situation, such as the home, analysing whole body movement using machine learning methods^25–27^. This method is based on the mechanical effect of blood ejection from the heart on hole body posture. More recently, methods allowing to extract HR at a distance from human subjects have been developed based on video image processing^28–30^. Imaging photoplethysmogram (iPPG), detects, as is the case for PPG, variation of skin light absorption/reflection properties^31–33^. Indeed, heartbeat induces blood flow in all the body including the face skin. This results in a change in the skin reflectance^34–38^. These changes in human skin reflectance can be tracked from webcam^39,40^ or smartphone^41^ video quality images associated with ICA signal processing techniques.

These methods appear highly relevant to non-human primate research and welfare, as cognitive processes, emotional states or welfare indicators such as stress can only be inferred by indirect measures. However, one of the major challenges in this context is the fact that changes in skin reflectance are more difficult to detect in monkeys due to reduced glabrous facial skin surface. In spite of this limitation, Unakafov et al.^42^ have successfully applied HR tracking in awake macaques in combination with discrete Fourier and wavelet transform based iPPG. While this study is of great interest, it falls short of two objectives that are useful to behavioural and experimental studies in non-human primates. First, Unakafov et al.^42^ do not address IR video-based hear rate estimation, although for example, a lot of neuroscience studies require video recording in light controlled environments. Second, they do not evaluate the sensitivity of their method in the awake behaving monkey and its ability to track subtle changes in heart rate, for example during the processing of emotional stimuli.

Here, we present an alternative indirect HR tracking method in the monkey, using Eulerian Video Magnification (EVM)^30^. A major advantage of this approach relative to iPPG is its resilience to subject motion^29^. We have further associated EVM video extraction with wavelet transformation based analyses, that has also been shown to be motion tolerant on poor quality human webcam video data^43^. In a first step, we show that EVM-based HR tracking has a high temporal coherence with ground truth ECG data, whether extracted from RGB video images or IR video images (that are often used in neuroscience experimental protocols) and that EVM-based HR estimate is very close to ECG-based HR estimate. For both types of video quality, we show that temporal coherence between EVM-based heart tracking and ground truth ECG is not significantly different between humans and monkeys. Last, we describe the dependence of temporal coherence between EVM-based heart tracking and ground truth ECG on the localization of the specific facial region EVM is performed onto. In a second step, we apply EVM-based heart tracking to the awake monkey and we show that this measure allows to identify consistent HR changes following the presentation of conspecific emotional voices or faces. Overall, we believe that this method can be generalized as a tool to track HR of the awake behaving monkey, for ethological, behavioural or welfare purposes.

## Material and methods

### Subjects

#### Monkeys

Three rhesus monkeys (*Macaca mulatta*) participated at this study. They were aged between 9 and 17 years (2 males: monkeys T and S, 1 female: monkey Z). The project was authorized by the French Ministry for Higher Education and Research (project no. 2016120910476056 and 2015090114042892) in agreement with the French implementation of European Directive 2010/63/UE. This authorization was based on the ethical evaluation by the local Committee on the Ethics of Experiments in Animals (C2EA) CELYNE registered at the national level as C2EA number 42 and the National Ethics of Experiments in Animals board of the French Ministry of Higher Education, Research and Innovation. In order to meet the ethical requirement of reduction, following the recommendation of the ethical committee, we describe our finding and perform our statistical tests in two monkeys.

#### Humans

Two human participants were included in this study and were covered by a broader project authorization (ID RCB 2018-A03438-47).

### Monkey anaesthesia

Monkeys were lightly anesthetized with Zoletil (Tiletamine-Zolazepam, Virbac, 10 mg/kg) so as to avoid head movements. During the video and ECG acquisitions, monkeys were gently resting on their side, under constant physiological monitoring.

### Monkey Behavioural task

During these sessions, monkeys were awake and sat in a sphinx position in a plastic monkey chair and head restrained to avoid movement. They faced a screen (1920 × 1200 pixels and a refresh rate of 60 Hz) placed 54 cm from their eyes on which stimuli were presented by an experimental control and stimulus delivery software (EventIDE). The auditory stimuli were displayed by a Sensimetrics S14 insert earphones and set up. Monkeys were required to fixate the centre of the screen all throughout the recording blocks. Eye fixation was controlled thanks to an Eyelink video eye tracker (EyeLink). Recording blocks consisted in 2 alternations of 16 s of fixation and 16 s of emotional stimuli presentation (alternations of 450 ms stimuli of the same sensory and emotional category, differing in specific identity). In one type of recording blocks, fixations alternated with highly emotional auditory content (screams). In a second type of recording blocks, fixations alternated with highly emotional visual content (4° × 4° aggressive faces). In total, 54 fixation to scream transitions (30 for monkey T and 24 for monkey S), and 57 fixation to aggressive faces (30 for monkey T and 27 for monkey S) were collected. The auditory stimuli and part of the visual stimuli were recorded in Cayo Santiago, Puerto Rico and kindly produced by Marc Hauser. The other visual stimuli were created in our own lab.

### Electrocardiogramm recordings

Electrocadiographic signal (ECG) was recorded thanks to a BiopacSystem. In humans, two electrodes were placed on the subject’s thoracic cage, on each side of the heart, while the reference electrode was placed on the abdomen, close to the stomach.

In macaques, two electrodes were placed on the subject’s thoracic cage, on each side of the heart, while the reference electrode was placed close to the groin on a shaved skin. ECG signal was recorded at a frequency of 2 kHz, in order to have a well-defined QRS waveform.

### Video recordings

We used a USB camera with a variable framerate (maximum frame rate of 30 frames per second), and a spatial resolution of 640 × 480 pixels. The camera had two working modes depending of light intensity. At high light intensity level, the camera was a color (RGB) device. At low light intensity level, the camera was an infrared (IR) device.

When comparing EVM-based HR tracking to ground truth ECG HR estimation in monkeys (monkey T and monkey Z), the animals were anesthetized and laying on their side. The video recording was targeted to the face. When comparing EVM-based HR tracking to ground truth ECG HR estimation in humans (subject H1 and H2), subjects were requested to gently and steadily gaze at the camera and video recording were targeted to the face. For each monkey and human subject, we collect ten minutes of videos in full light (RGB mode) or in the dark (IR mode).

When analysing the effect of emotional stimuli on EVM-based HR tracking, we recorded IR videos while monkeys T and S were performing the above described task.

ECG and video time series were synchronized using the AcqKnowledge software. Specifically, the AcqKnowledge software was used to record the ECG signal and send start and stop synchronization triggers to the video recording system.

### HR extraction from recorded video

#### Definition of Region of Interest (ROI)

**Figure 1:**
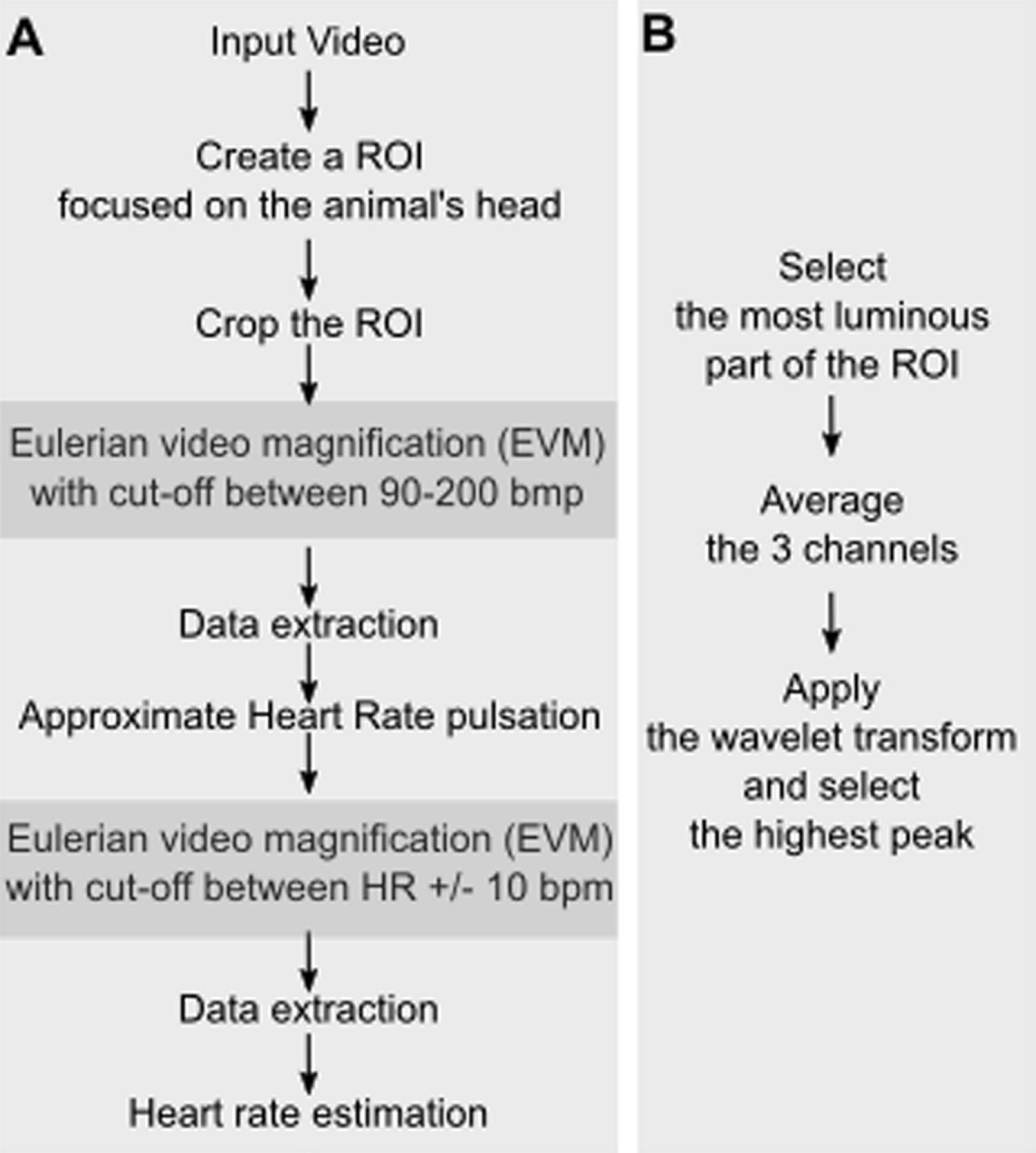
(**A**) *HR video extraction pipeline.* A Region of interest (ROI) is defined on the video input, ideally placed in a hairless skin region, such as the face. The video is cropped around this ROI selection and serves as input to the Eulerian video magnification (EVM) algorithm (see text for specifications). The result is a video in which luminosity changes due to HR are enhanced. A first HR approximation is extracted from this video (as described in B). In order to obtain a more precise HR estimate, this approximated HR is used as a parameter for a second EVM processing round. (**B**) *HR approximation.* Average time series are extracted from the ROI pixels of highest luminosity, for each color channel, and their frequency power profile is extracted using a wavelet transform. Peak frequency is taken as the estimate of HR.

In order to optimize HR extraction, the first processing step involves defining, in the recorded video a region of interest (ROI) to feed in the rest of the processing pipeline (Fig. 1A). Because subsequent processing involves estimating variations in skin luminosity, ROIs should be placed on the face. The optimal location on the face is further discussed in the result section. The video is cropped around this ROI and the output is fed into a first EVM processing step.

#### Eulerian Video Magnification –step one

The Eulerian video magnification algorithm or EVM^30^ allows to magnify variations in frame to frame video information and can thus be used amongst other things to amplify skin colour variations due to blood circulation. It involves both a spatial and a temporal processing such that any variation in pixel properties through time and space is amplified. This can be modelled as follows: on a 1D signal, let I(x,t) be the intensity of the signal at pixel *x* and sapling time *t*. The displacement function, δ(t), represents at a given time the distance of the pixel from its original position. Thus the intensity of the pixel of interest can be written as

$${\text{I}}\left( {{\text{x}},{\text{t}}} \right) = {\text{f}}\left( {{\text{x}} +\updelta \left( {\text{t}} \right)} \right)$$

where at the initial condition *t* = 0, the intensity of the pixel *x* takes the value:

$${\text{I}}\left( {{\text{x}},0} \right) = {\text{f}}\left( {\text{x}} \right)$$

The EVM process consists in adding an amplified displacement function to the original intensity signal of gain α. Considering that the signal is within the selected range of frequencies of an ideal band-pass filter, the intensity signal generated by the EVM is defined as:

$${\hat{\rm I}}\left( {{\text{x}},{\text{t}}} \right) = {\text{f}}\left( {{\text{x}} + \left( {1 + \upalpha } \right)\updelta \left( {\text{t}} \right)} \right)$$

Outside the selected frequency range, the intensity signal is set to identity: Î = I. This results in the selective amplification of movement information in the 1D signal within the frequency range of interest. This procedure can be generalized to a 2D signal such as a video. The input video is decomposed into several spatial frequency bands. The same temporal filter is applied to all these bands. The most relevant spatial frequency band to the signal of interest (here, HR detection) is amplified and the result of this amplification is added to the initial video signal. Amplification factor (α) and temporal filtering parameters are hand-optimized by the experimenters to maximize the identification of the signal of interest.

Specifically, we use the EVM functions for Matlab (https://people.csail.mit.edu/mrub/evm/), and run it on our ROI-cropped video, defining a spatial filtering by a Gaussian blur and a down sample, as well as a temporal filtering by a band pass filter matching the expected range of HR estimates (e.g. 90 to 200 bpm^12,44,45^). The output of this first EVM processing round is a video in which luminosity changes due to HR are enhanced (Fig. 1A).

#### HR approximation

The ROI pixels with highest frequency power are further extracted, all video colour time series are averaged and a wavelet transform is used to select frequency with highest peak in these selected pixels (Fig. 1B).

#### HR estimation—step two

In order to obtain a more precise estimate of HR in time, the EVM is run onto the selected ROI-cropped video a second time, and a band pass temporal filtering better matching the individual subject HR range is applied (Fig. 1A). A second HR estimation is run as described in Fig. 1B and the HR approximation paragraph above.

### Signal processing

**Figure 2:**
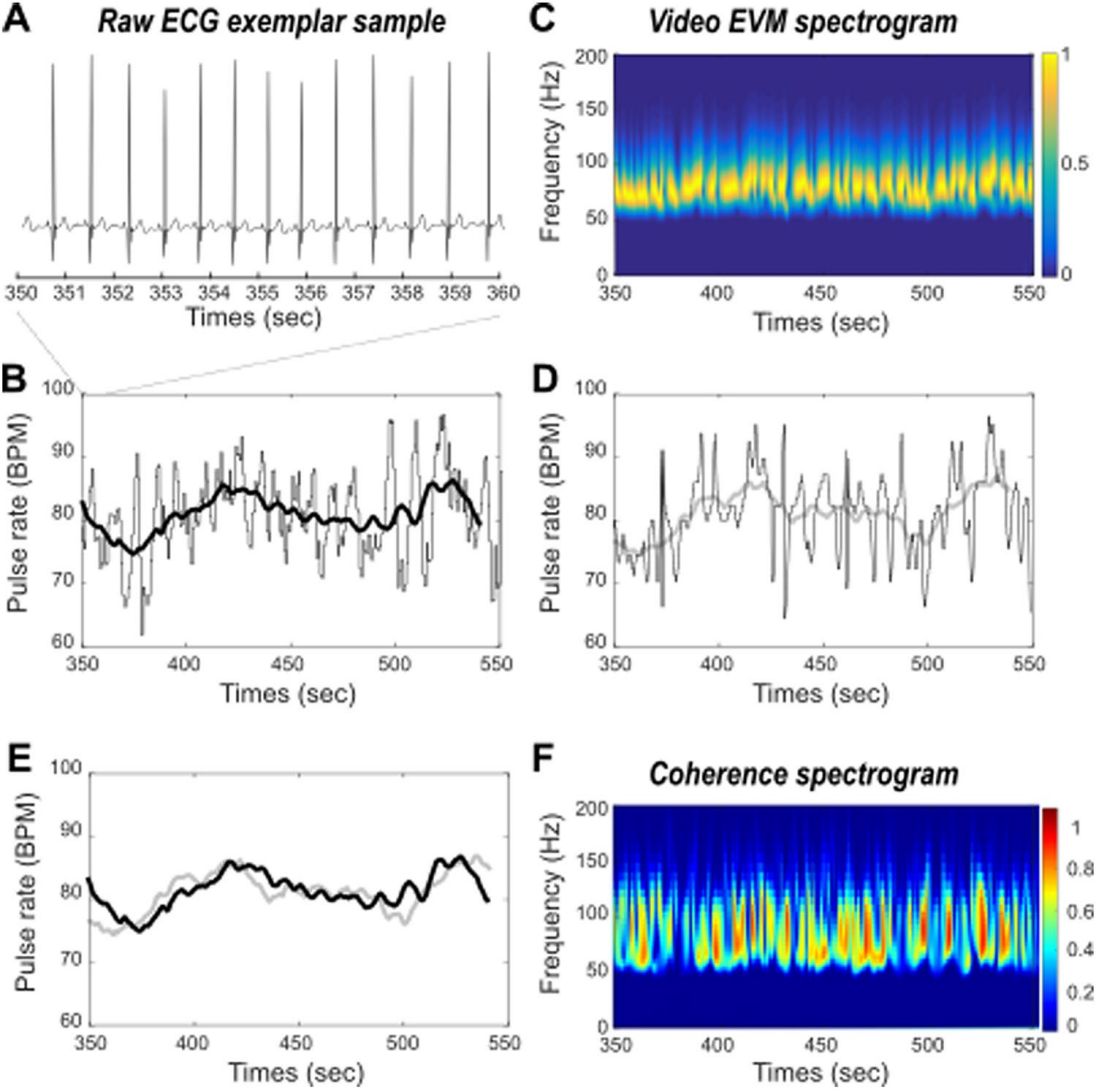
Comparing ECG and EVM HR estimates. **(A**) Raw ECG exemplar sample. (**B**) ECG inter-peak interval estimate (gray) and pulse rate running average (black) in time. (**C**) Time frequency spectrogram of EVM processed video data. Highest power frequency band (yellow) corresponds to the HR estimate. (**D**) EVM peak frequency estimate (gray) and corresponding pulse rate running average (light gray) in time. (**E**) Overlay of ECG (black) and EVM (light gray) based running average pulse rates estimates in time. (**F**) Coherence between ECG and EVM HR estimates.

*ECG-based HR* Inter-peak intervals were extracted from the raw ECG signal and pulse rate were estimated in time by computing a running average over the inter-peak times series (Fig. 2b, averaging window size = 20 s).

*EVM-based HR* Instantaneous HR estimates are defined as maximal power frequencies at each time step of the wavelet transform. EVM-based HR is estimated in time by computing a running average over the instantaneous HR times series (Fig. 2c, averaging window size = 20 s).

**Figure 3:**
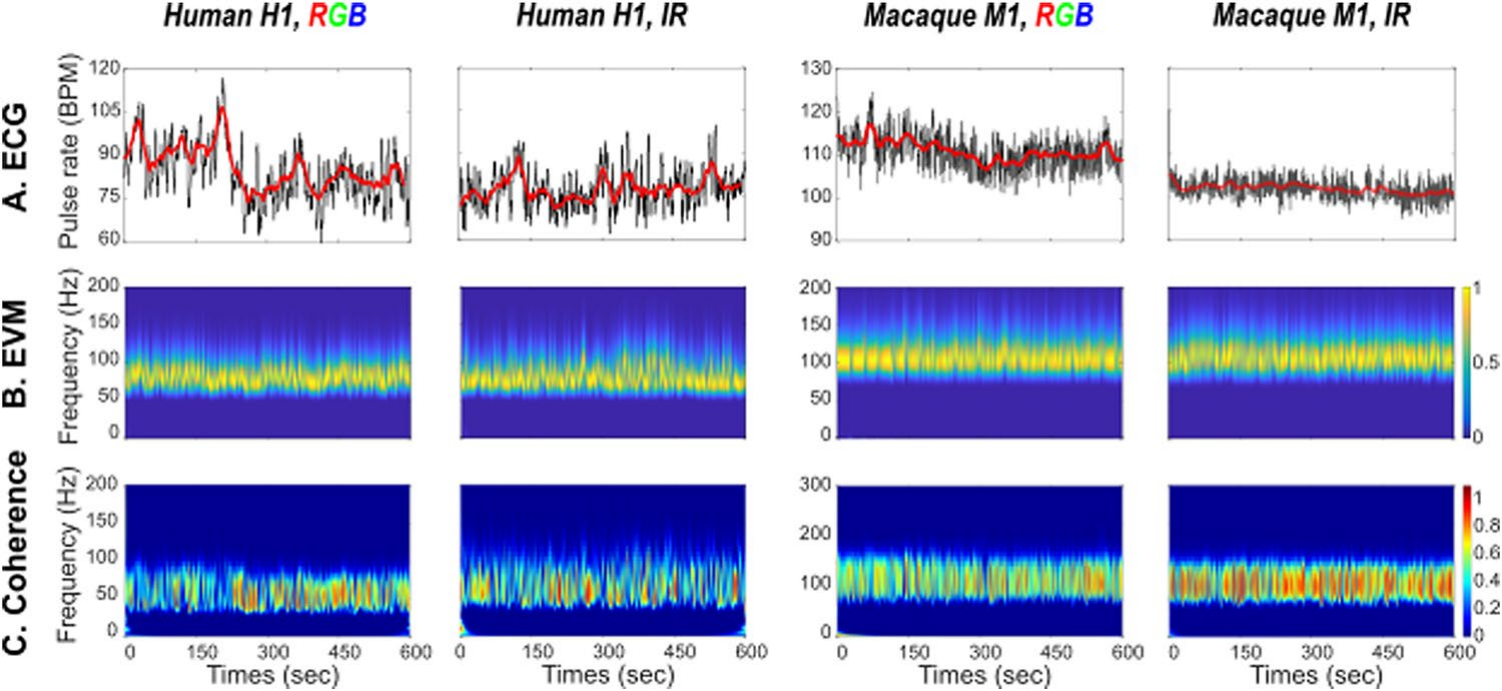
HR estimation in humans (panels 1 & 2) and monkey (panels 3 & 4) based on RGB (panels 1 & 3) and IR (panels 2 & 4). (**A**) ECG HR ground truth. (**B**) EVM HR estimate. (**C**) Coherence between ECG and EVM HR estimates.

*ECG–EVM temporal coherence* In order to statistically assess the extent to which ECG-based and EVM-based HR measures co-vary in time, we perform a wavelet coherence analysis that estimates instantaneous coherence in time and for all frequencies of interest (Fig. 3). Reported temporal coherence is the average of maximum temporal coherence in time, across all frequency ranges. The wavelet coherence improves the time–frequency localization of spatial correlation patterns. It is also particularly well suited to non-stationary and noisy physiological data^46^.

*Stimulus triggered changes in EVM-based HR* EVM-based HR is estimated for each run and each monkey the pulse rate along the entire recording blocks. HR time series are extracted around the onset of the stimulus of interest (i.e. scream or aggressive face, [− 16 s 16 s]) and realigned to stimulus event presentation (0 s time reference). Individual time series are baseline corrected ([− 13 − 3 s]) and mean + /− s.e. is computed in time. Pre ([− 13 − 3 s]) and post-stimulus ([3 13 s]) HR estimates are compared using a non-parametric Wilcoxon test.

### Ethical standards statement

#### Monkeys

Three rhesus monkeys (*Macaca mulatta*) participated at this study. They were aged between 9 and 17 years (2 males: monkeys T and S, 1 female: monkey Z). The project was authorized by the French Ministry for Higher Education and Research (Project No. 2016120910476056 and 2015090114042892) in agreement with the French implementation of European Directive 2010/63/UE. This authorization was based on the ethical evaluation by the local Committee on the Ethics of Experiments in Animals (C2EA) CELYNE registered at the national level as C2EA number 42 and the National Ethics of Experiments in Animals board of the French Minsitry of Higher Education, Research and Innovation. All experiments on non-human primates were performed in accordance with relevant guidelines and regulations.

#### Humans

Two human participants were included in this study and were covered by a broader project authorization (ID RCB 2018-A03438-47). All individual data was anonymized. All human experiments were performed in accordance with relevant guidelines and regulations. All experimental protocols were approved by the CNRS (research institution acting as promotor) and the CPP-Sud-Est (acting as licensing committee). Informed consent was obtained from all participants.

#### Analysis

All statistical analyses are non-parametric Wilcoxon rank-sum tests that do not assume normally distributed data. This tests the hypothesis according to which the medians of two independent data distributions are close to each other. Such non-parametric statistical tests are usually considered as more stringent then parametric statistical tests. The effect size Cohen's *d* was calculated by dividing the mean difference between HR before and after the stimulus by the global standard deviation during the run. When the Cohen's *d* is equal to 0.2, the effect is considered as small effect, 0.5 as medium and 0.8 and more as large.

## Results

### Comparing EVM-based HR estimation to ground truth ECG

In order to validate the EVM-based HR measure, we compared the inter-pulse signal estimated using the EVM approach (Fig. 1) to ground truth ECG-based inter-pulse estimation. Figure 2A represents a sample of our ground truth recording measure in a human subject, and the corresponding inter-peak estimation (Fig. 2B, gray) and pulse rate running average on a longer recording period (Fig. 2B, black). Figure 2C represents, during the exact same time period, the frequency over time, the EVM-based time frequency output of our EVM processing pipeline (Fig. 1). HR induced rhythmic changes in the recorded video flow are clearly observed in the 70 to 90 Hz frequency range (Fig. 2C, yellow saturation epochs). At each time point, EVM-based instantaneous HR peak frequencies are extracted resulting in an EVM-based pulse rate estimation in time (Fig. 2D, gray, running average, light gray). The ECG-based (Fig. 2E, back) and EVM-based running averages (Fig. 2E, gray) strongly co-vary in time. In order to statistically assess the extent to which ECG-based and EVM-based HR measures co-vary in time, we perform a wavelet coherence analysis that estimates instantaneous coherence in time and for all frequencies of interest (Fig. 2F). This statistics computes, at each time point of the time series, and each frequency, the degree of coherence between the two signals of interest. On this specific recording, average temporal coherence between ECG-based and EVM-based HR estimates is 0.5 (s.d. = 0.22).

### EVM-based HR estimation performs better in monkeys than in humans, whether using color (RGB) or infrared (IR) videos

EVM-based HR estimation has already been validated with human RGB data^30^. Here, we confirm that HR can be estimated from human RGB (Fig. 3, first column) as well as IR videos (Fig. 3, second column). We further show that EVM-based HR estimation can also be achieved from anesthetized macaque RGB (Fig. 3, third column) as well as IR videos (Fig. 3, fourth column). For each type of video, each column represents ECG-based inter-pulse estimation and corresponding running average (upper row), normalized EVM-based pulse rate power estimation (middle row) and temporal coherence between ECG-based and EVM-based HR estimates (lower row).

Table 1 further summarizes pulse rate statistics for each type of recording. Differences between ECG and EVM-based HR estimations range between 0.07 BPM and 8.28 BPM (mean = 0.85, s.d. = 4.01). Both methods reach very similar signal variability (mean s.d.: ECG: 2.9275; EVM: 2.6575). Average coherence between the two signals is 0.51 (s.d. = 0.08). Importantly, coherence between ECG-based and EVM-based HR estimates tend to be higher in monkeys (mean = 0.5763, s.d. = 0.05) than in humans (mean = 0.4554, s.d. = 0.05, unilateral Wilcoxon, p = 0.06, Effect size: Cohen’s *d* = 2.418). Overall, this is thus evidence for the fact that EVM can be used to provide a reliable non-invasive measure of HR estimation in the anesthetized preparation.

**Table 1:** Pulse rate estimation statistics from ECG and EVM time series and corresponding temporal coherence.

		ECG-based pulse rate		EVM-based pulse rate		Coherence coefficient
		Mean	S.D.	Mean	S.D.	
Human 1	RGB	84.10	5.64	80.83	2.9	0.48
	IR	77.95	2.9	77.88	2.3	0.4656
Human 2	RGB	84.76	4	76.48	3.3	0.377
	IR	81.35	2.88	82.08	4.61	0.499
Monkey 1	RGB	109.5	1.74	105.79	1.49	0.554
	IR	102.5	0.89	105.5	1.88	0.66
Monkey 2	RGB	178.35	1.94	182.26	2.95	0.5676
	IR	167.16	3.43	168.05	1.83	0.5236

### Effect of ROI selection on EVM-based HR estimation

**Figure 4:**
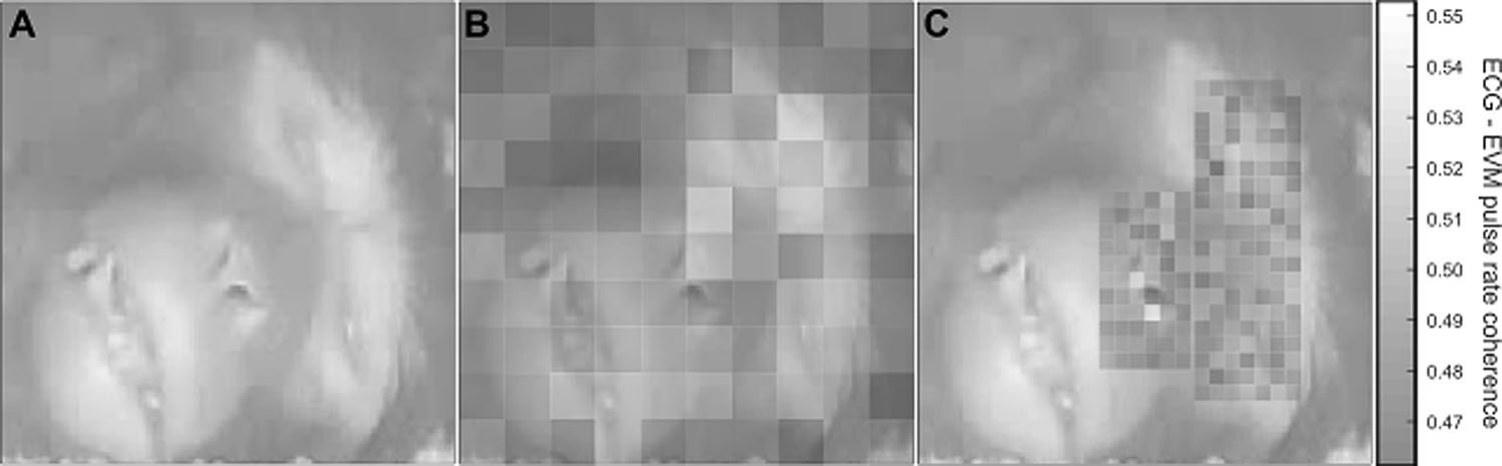
Effect of ROI size and localization on ECG-EVM pulse rate coherence. (**A**) Reference monkey face on which is superimposed ECG-EVM coherence maximum power based on a 10 × 10 pixel matrix covering the entire face (**B**) or smaller pixels covering the eyes and muzzle (**C**). Note that B and C share the same color scale.

During the EVM signal analysis, a ROI must be defined in order to optimize HR extraction. This ROI is usually placed in the most luminous part of the video, in order to record the luminosity variabilities as precisely as possible and thus have the best EVM-based HR estimation. (see Fig. 1B). Although coherence between ECG and EVM-based HR estimations is always higher than 0.45, it can reach up to 0.55 when adequately placed on the face. Figure 4A represents the initial video frame recorded in an anesthetized monkey. Figure 4B represents coherence between ECG and EVM-based HR estimations computed over 10 × 10 independent ROIs covering the video frame presented in Fig. 4A. Coherence is overall higher in the glabrous skin parts of the face (eyes and eye lids, snout and mouth), although important variations can still be observed between different parts of this glabrous skin. The skin around the eyes and the snout appear to be most informative in relation with heart-rate extraction. Focusing on the face regions (Fig. 4C) and defining 270 smaller pixels in this region produces local pixels of maximal coherence at the bottom of the snout. However, on average, these smaller voxels do not produce higher coherence between ECG and EVM-based HR estimations than larger voxels as presented in Fig. 4B.

### EVM-based HR estimation tracks changes in HR in the awake behaving monkey

**Figure 5:**
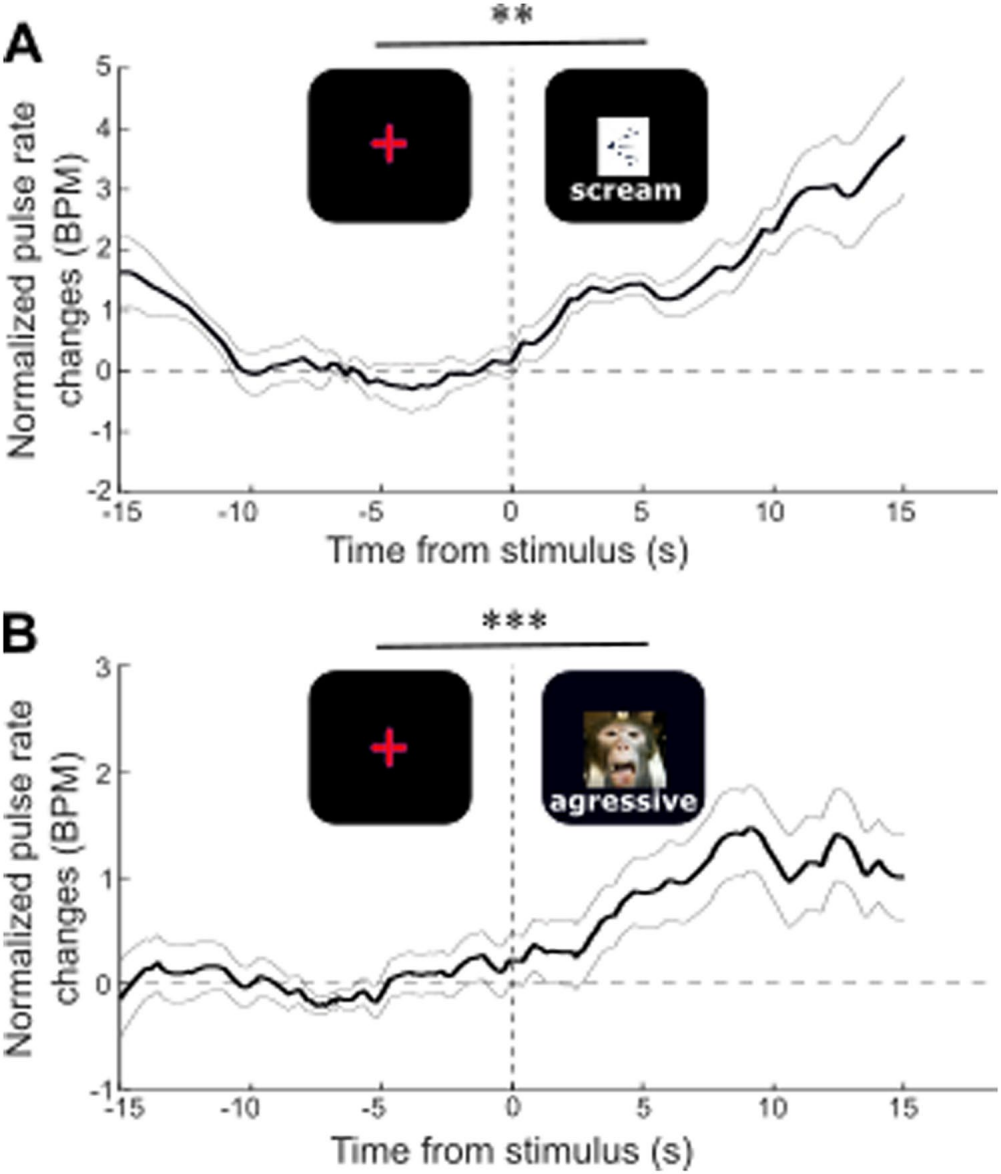
EVM HR estimate modulation (mean + /− s.e.) by (**A**) monkey screams (Wilcoxon test comparing pre-stimulus [− 400 − 100 ms] and post-stimulus [100 400] epochs, *p* = 0.03) and (**B**) monkey aggressive faces (*p* = 0.001).

The main objective of this paper is to provide a non-invasive alternative to ECG and pulse oximeter HR tracking method for monkeys during typical behavioral tasks, with no behavioral training requirements. During such tasks, HR measures have been shown to co-vary with cognitive^47,48^ or emotional processes^13^. A non-invasive HR tracking alternative thus needs to be able to track subtle changes in HR while monkeys are actively performing a task. Here, we required two monkeys to maintain eye fixation on a central cross for reward, while stimulating them with either monkey screams (Fig. 5A, auditory negative emotion) or with a static aggressive monkey picture (Fig. 5B, visual negative emotion). Meanwhile, we recorded their faces thanks to an IR video system. The videos were EVM-processed as described in Fig. 1, placing a ROI between the two eyes, at the base of the snout, allowing to obtain an optimal estimate of HR. Then, we synchronized the latter with events, characterized by the sensory stimuli and corrected it with respect to the pre-event baseline (baseline corrected), such thus we could report the HR changes induced by stimulus presentation, irrespective of other possible modulatory effects. Both the monkey screams (Fig. 5A, pre-post comparison, Wilcoxon p = 0.03, Effect size: Cohen’s *d* = 0.3943) and the aggressive monkey faces (Fig. 5B, *p* = 0.001, Effect size: Cohen’s *d* = 2.05) induce a small but systematic change in HR. The onset of these systematic changes are of the order of a few seconds and are compatible with the reported latency of HR changes during emotional processing^13^.

## Discussion

Overall, we demonstrate that EVM combined with wavelet transform analyses allows to reliably extract HR estimates from both RGB and IR videos of both anesthetized and awake macaque monkeys. These HR estimates are directly comparable to ground truth ECG HR estimate, as they have the same temporal stability and show a high temporal coherence with these reference signals. These EVM-based HR estimates also show a higher similarity with ECG in monkeys than in human subjects (max difference in BPM: human: 8.28 BPM; monkeys: 3.91 BPM) probably due to the fact that monkeys were under anaesthesia while human subjects were awake. Irrespective of anaesthesia and quite remarkably, coherence between EVM-based and ECG-based HR estimates tended to be higher when extracted from monkey videos than from humans. While this could still be due to improved stability of the face in the video stream in the anesthetized preparation, it still noteworthy, as human faces have more glabrous as well as a thinner skin, thus allowing for a better capture of superficial vascular changes. In any case, reporting this temporal coherence measure is crucial as it indicates that EVM-based HR estimation is sufficiently sensitive to track actual physiological variation of HR in time. These EVM-based HR estimates compare to those reported in a previous study extracting monkey HR from videos using imaging photoplethysmogram^42^. Importantly, and in contrast with this previous study, EVM-based HR estimates can be reliably obtained both from RGB and IR videos.

Our present work addresses one crucial aspect of highest relevance to behavioural and cognitive studies in non-human primates, namely HR estimation in awake behaving monkeys. Here, using our EVM-based HR estimation method, we reproduce the observation that emotional sensory stimuli, whether it be auditory or visual, induce HR changes in monkeys^13^. Reported HR changes can thus be specifically associated to stimulus emotional informational content rather than to visual stimulus presentation. This is an important point as local luminosity changes due to for example visual stimuli presentation have been shown to affect plethysmographic signals^49^ but not ambient light^50^. Indeed, HR has been shown to be modulated in a variety of tasks: emotional or valence tasks^13,51–53^, attentional tasks^48,54–56^ as well as learning tasks^47^. Tracking this physiological parameter non-invasively, is methodologically easier, brings no stress and thus improves animal welfare while experimenting, saves training time and minimizes possible measure biasing factors (for example when animals are training to accept pulse oximetry measures). In addition, HR measure has been shown to covary with pupil dilation, typically controlled by the autonomic nervous system^57^. Pupil dilation has been shown to vary as a function of reward^57,58^, surprise^59^, vigilance for social distractors^60^, arousal^61^ and systemic pharmacological neuromodulation^5^. Last, HR can be a crucial parameter to assess behaviour following a drug induction^62^ or a lesion^15^. Overall, this ability to track changes in HR measure in an experimental context, that we demonstrate here, is thus of major implications to behavioural, cognitive and neuroscience experiments in awake macaque monkeys. This method is less expensive and requires no training. It can thus now be recommended and implemented as a control measure in most such awake macaque experiments.

In order to improve HR detection from videos, a recent paper suggests to use neural networks to detect and enhance the ROI from which the signal is extracted^63^. This technique can be implemented to detect moving animals in open fields or working animals in less restrained environments than ours, provided a high-resolution fast frame rate camera is used. This would be an important add-on to our work as HR and HR variation in monkeys in their home cage, during their time in the experimental setup or in open zoo spaces is considered as a good health state and stress marker^12,44,45^. For example, Hassimoto et al.^64^ tracked the first 3 months of acclimation of rhesus monkeys in a new laboratory environment and they demonstrate a decrease of HR following this stressful period^64^.

In summary, the method we describe here is an easy and reliable non-invasive alternative for HR estimation both in anesthetized and awake monkeys. It has several notable advantages. It is very easy to set up as it only requires a decent recording camera and a computer to implement the processing pipeline. It is stable over time and does not require any specific training from the animal due to its absence of body sensors. It works in a classical behavioural task environment and is resilient to luminosity changes. Its major disadvantage lies in the fact that it doesn’t, as it stands, allow real-time tracking of HR or the tracking of HR in freely moving subjects. Once these two limitations are overcome, the method will be usable during veterinary or health visits whether under sedation or light anaesthesia, under minimal contention condition, or even in home cage or open field environments.

The generalization of this method to experimental and ethology animal facilities can be considered as a contribution to animal welfare. During actual cognitive, behavioural or neuroscience experiments it can be considered as a refinement measure as well as a crucial physiological control parameter to include in the data analyses or in the framework of data sharing consortia^65,66^. By extension, we expect that this method can also be applied on young infants in neurodevelopment studies, where pulse oximetry HR tracking can turn out to be extremely challenging.

## Conclusion

Overall, the non-invasive video-based HR extraction method described here generalizes to experimental situations that have not been addressed by previous studies. First, it successfully extracts non-human primate HR estimates both from RGB videos, as previously achieved by others, as well as from IR videos. Second, we show that this method is very robust to small head movements, successfully tracking HR in the awake behaving monkey. Third, we show that this method is sufficiently sensitive to track HR variations during the exposition of non-human primates to emotional visual and auditory stimuli. It thus represents a non-invasive low cost and easy to implement HR tracking method that can be used in multiple anesthetized and awake monkey behavioural, veterinary and experimental set-ups. Association with automated face detection algorithms is further expected to generalize this method to open-field situations, thus representing a real breakthrough in the study of monkey behaviour and well-being.
